# Remembering Ada L. Steininger

**DOI:** 10.1016/j.jacbts.2024.05.004

**Published:** 2024-06-24

**Authors:** Douglas L. Mann

**Figure:**
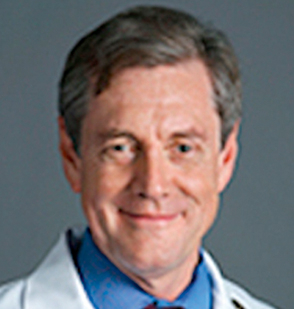

I am the current Ada L. Steiniger Professor of Cardiology at Washington University. The Steinger professorship was established at Washington University in 2021 by the Dean of the School of Medicine, Dr David Perlmutter, using an estate gift provided by Ms Steiniger to support research in heart disease and cancer. When I first learned that I would receive the Steininger endowed chair, I wanted to reach out and say thank you to the Steiniger family for supporting my research, only to learn that all of the members of her family had died, and that there were no heirs. So, there was no one for me to thank. To this end, I wanted to dedicate this Editor's Page to a woman whom I have never met, but who has touched my life in a meaningful way.

Ada L. Steiniger, nee Locatell, died on November 27, 1971, in Saint Louis, Missouri. She was predeceased by her husband, Mr Walter Steiniger, who passed away at age 51 years while the couple was living in the Chicago area. The couple did not have any children. At some point after her husband’s death, Ms Steiniger decided to move to St. Louis to live with her brother, Arthur Locatell, who was a native St. Louisan. Arthur attended Washington University and graduated in 1923. Arthur began his career as a bookkeeper at the Tower Grove Bank in 1911 and rose to become president of that bank in 1929. He held that position until 1956, at which time he was elected as Chairman of the Board of Directors. Arthur's interest in health care likely stemmed from the time he was director of the Bethesda General Hospital, a community hospital that served the St. Louis community from 1901 to 1944. He was also a director of the St. Louis Altenheim, which has been in existence for 100 years and currently serves as a senior living facility. Mr Locatell passed away in 1967 and was survived by Ada, who was his only sibling.

Unfortunately, the information I have provided in the previous text is all that I was able to find about Ms Steininger after reading a few short newspaper clippings. Digital footprints were just not a thing in the 1970s. In the absence of more definitive information, I mused that Ada must have had a rich and rewarding life during her time in St. Louis. Surely, she became a St. Louis Cardinals baseball fan after moving to town. I also imagined that her commitment to supporting heart research must have come about after she and her brother Arthur discussed the latest developments in heart research, including the Nobel prize in Medicine, which was awarded to Werner Forssmann, André Cournand, and Dickinson Richards in 1956 for developing the technique of cardiac catheterization, or the equally exciting news in 1960 that Dr Robert H. Goetz had performed the first successful coronary artery bypass surgery at Albert Einstein Hospital in the Bronx, New York.

Although Ada did not have any heirs, her legacy will live on through the generations of Ada L. Steiniger Professors of Cardiology who will benefit from the support of her enduring gift. As a final tribute to the kind of person that Ada L. Steininger was, I learned that she expressed a preference for donations to be made to the St. Louis Heart Fund in lieu of sending flowers for her funeral. She was in death as she was in life: someone who cared deeply about improving the lives of people afflicted with cardiovascular disease.

